# Job satisfaction of midwives working in a labor ward: A repeat measure mixed-methods study

**DOI:** 10.18332/ejm/145494

**Published:** 2022-02-14

**Authors:** Susanne Grylka-Baeschlin, Regula Aeberli, Barbara Guenthard-Uhl, Barbara Meier-Kaeppeli, Vanessa Leu-tenegger, Thomas Volken, Jessica Pehlke-Milde

**Affiliations:** 1 Research Institute for Midwifery Science, ZHAW Zurich University of Applied Sciences, Winterthur, Switzerland; 2 Division of Women’s Health and Newborn Care, Department of Obstetrics, University Hospital of Zurich, Zürich, Switzerland; 3 Research Institute for Health Sciences, ZHAW Zurich University of Applied Sciences, Winterthur, Switzerland

**Keywords:** occupational satisfaction, skill shortage, work-related stressors, midwife-led models of care, debriefing, implementation

## Abstract

**INTRODUCTION:**

Job satisfaction of midwives is important to prevent skill shortage. Those working in midwife-led models of care work more independently and have more responsibility. No previous study investigated if a self-initiated and self-responsible project could enhance job satisfaction of midwives working in a medicalled maternity unit. The aim of this study was therefore to assess job satisfaction before and after the implementation of such a project.

**METHODS:**

This is longitudinal observational study at three time points using quantitative and qualitative methods. A total of 43 midwives working in a Swiss labor ward participated in the online surveys and in the focus group discussions. The surveys comprised questions from validated instruments to assess job satisfaction. Descriptive and multivariable time series analysis were used for quantitative and content analysis for qualitative data.

**RESULTS:**

Adjusted predicted scores decreased between t_0_ and t_1_, and subsequently increased at t_2_ without reaching baseline values (e.g. ‘professional support subscales’ between t_0_ and t_1_: (0.65; 95% CI: 0.45–0.86 vs 0.26; 95% CI: 0.08–0.45, p=0.005) and between t_0_ and t_2_ (0.65; 95% CI: 0.45–0.86 vs 0.29; 95% CI: 0.12–0.47, p=0.004). Focus group discussions revealed four themes: ‘general job satisfaction’, ‘challenges with the implementation’, ‘continuity of care’ and ‘meaning for the mothers’. Midwives perceived the additional tasks as stressors.

**CONCLUSIONS:**

The implementation of new projects might enhance work-related stress and consequently have negative impacts on job satisfaction in an early phase. Heads of institutions and policy makers should recognize the needs of support and additional resources for staff when implementing new projects.

## INTRODUCTION

Job satisfaction of midwives depends on their working environment including possibilities to take responsibility and build relationships with clients. Studies have shown that positively experienced workplace qualities and occupational satisfaction enhance the chances that midwives as well as other health professionals remain in their positions for longer, and consequently prevent skill shortage^1-4^. In contrast, work-related stress was found to be negatively associated with job satisfaction but positively with the intention to leave the work place or even the profession^5,6^.

Job satisfaction is a complex concept and is not uniformly defined^7^. It is related to a person’s overall evaluation of whether his or her job is favorable or not^8^. Factors such as working conditions, compensation, social relationships, work-related demands and perceived long-term opportunities are related to job satisfaction. This in turn has implications on commitment to the organization or institution and staff turnover^7,9^. Similarly, job satisfaction of midwives was found to be associated with their working relationship with colleagues, supervisors’ clients, accomplishing high quality of care, manageable working hours and a good salary^1,10^. Professional activities of midwives have also been shown to cause substantial work-related stress such as the incompatibility of family life and work, demarcation problems and lack of appreciation of their work^5,11,12^. A German study including more than 2000 participants demonstrated that a substantial number of midwives were unsatisfied with the recognition of their work (43%) and with the compatibility of family life with work (33%)^5^. More than a third of midwives (39%) were not satisfied with their general working conditions and more than a fifth (22%) would not or rather not choose their profession again. More than 40% of midwives did leave their profession according to a Swiss inventory^2^. Some studies showed differences in factors in relation to job satisfaction between hospital midwives and those working in primary care^11,13^. For hospital midwives, the most significant domains were working hours per week, workplace agreements, workload, the lack of recognition by medical staff, and total years of experience. For primary care midwives in contrast, social support at work, work demands, job autonomy, and compatibility of family life and work, were the most significant^11,13^.

More self-responsibility, providing higher quality of care and building deeper relationships with women due to continuity of care are reasons that midwives working in midwife-led models of care were found to be more satisfied than those providing traditional care^4,14-16^_._ Midwife-led care is characterized by being woman-centered and fostering physiological process as well as continuity of care^16,17^. While midwives are the lead care provider from pregnancy to the postpartum period in midwife-led models of care^16^, in many countries medically-led models are predominant.

As in many other countries, midwife-led models of care in Switzerland are rare^17-19^. Midwife-led births such as home births, birth center births and midwife-attended births in hospitals amount only to approximately 6.5% of all births in Switzerland^20,21^. In addition, there are increasing hospital-internal initiatives for midwife-led births, which currently cannot be quantified. Most midwives work shifts in obstetrician-led maternity hospitals^17–20^. They have limited opportunities to take responsibility, work independently and provide continuity of care. No previous study investigated if a self-initiated and self-responsible project, which enhances continuity of care from birth to the postpartum period could increase job satisfaction of midwives working in a medical-led maternity unit. The aim of this study was therefore to assess job satisfaction before and after the implementation of such a project.

## METHODS

In our reporting, we followed the STROBE guidelines for observational studies and the SPQR guidelines for qualitative studies^22,23^.

### Study design, setting and context

We conducted a prospective longitudinal observational study in 2018 using quantitative and qualitative methods. Data were collected at three time points, before, at two and at seven months after the implementation of a midwife-initiated and self-responsible project in a Swiss university hospital. The project consisted of telephone debriefing sessions of women being called approximately six weeks after birth by the midwife who provided intrapartum care. The project aimed to foster self-responsible work of midwives and to enhance continuity of care from birth to the postpartum period. It was initiated by a core team of eleven experienced midwives. A conversation guide was developed by a midwifery expert in collaboration with a psychologist to structure and advise the debriefing sessions. The conversations lasted approximately 15 minutes each and dealt with the following topics: women’s birth experience and processing, clarifying open questions and the need for a face-to-face session, wellbeing of the family and if necessary, contact details for counselling services. Midwives were trained before the implementation of the project and were expected to conduct one session every 2 to 2.5 workdays.

### Sampling

The study population included all midwives working in the labor ward of the university hospital and caring for childbearing women. The midwifery team consisted of 41 to 48 midwives at the different data collection time points. The exclusion criterion was being engaged exclusively with managerial responsibilities (n=1, all three time points) or being on maternity leave (n=2, third time point). A full census was targeted with a total of N=50 midwives who could participate at least at one time point. Thereof, n=43 midwives formed the study sample, of whom 19 completed all three questionnaires, 14 two questionnaires and 10 completed only one questionnaire. Response rates for the online surveys were 85.4% (n=35, first questionnaire), 75.0% (n=30, second questionnaire) and 66.7% (n=30, third questionnaire). Reasons why midwives did not participate in the survey could not be recorded due to the anonymous nature of the questionnaire. Participants of the three focus group discussions originated from the same study population and were all midwives working in the labor ward providing intrapartum care. Between five and seven midwives took part in the discussions. Study participants and the authors of the study all originated from Central and Eastern Europe and were all Caucasians.

### Data collection

Data were collected at three time points: before the implementation of the telephone debriefing sessions as well as at two and at seven months afterwards. At each time point, an online questionnaire was sent to all eligible midwives and a focus group discussion was conducted.

The online surveys were based on different validated instruments to assess job and occupational satisfaction^24-27^. In particular, the German version of the midwifery specific instrument to assess job satisfaction from Turnbull et al.^24,28^ was used. The instrument was previously translated into German and applied with independent midwifes in Switzerland^28^. Additionally, selected questions of the German version of the Copenhagen Psychosocial Questionnaire (COPSOQ)^29^, the Work Ability Index (WAI)^30^, self-translated questions of the domain ‘decision authority’ of the Leiden Quality of Work Life Questionnaire for Nurses (LQWLQ-N)^25^ as well as sociodemographic questions of the STRAIN-project were used^9^.

The semi structured interview guide for the focus group discussions was developed evidence-based^24-26,31^. The following themes were addressed: attitude toward the project, organizational aspects of the debriefing sessions, self-responsible work, continuity of care, client relationship, professional support, professional development, and general professional satisfaction.

### Data preparation and analysis

Quantitative data of the three online questionnaires were merged using an anonymized ID-code which was generated at the beginning of each survey. For categorical variables frequency and percentage are reported, and for metric variables mean, median, range and standard deviation were computed according to the distribution of data. Scores of validated instruments were calculated as proposed by the developer of the scales. Repeatedly measured categorical variables with more than two categories were compared using Skillings-Mack tests (Friedman test when there were missing data), because the data set was not complete due to loss of follow-up and new employees during the relatively long study period. Generalized estimating equation (GEE) models of the Gaussian family with robust standard errors and with log link in order to assess adjusted temporal trajectories of Instrument scores were used. Respective scores were adjusted for age, work years in the institution, number of women cared for per shift and workload. Confounders were chosen that were known from the scientific literature^13,32^ or showed the strongest bivariable associations with the outcome variables (p<0.25). We reported corresponding point estimates with 95% confidence intervals (95% CI). Statistical significance was established at p<0.05. We used Stata Version 13 (StataCorp, College Station, TX, USA) for all statistical analyses.

Focus group discussion were transcribed verbatim and analyzed using qualitative content analysis methods according to Kuckartz^33^. Deductive and inductive coding were applied, and codes were grouped into themes. Two coders were involved in the analysis process and disagreements were discussed and resolved by consensus. Sense of codes were reflected, and codes summarized into themes in an analysis meeting. Three researchers, including two midwifery researchers with knowledge of the work on a labor ward, were involved in this process to increase reflexibility, confirmability and transferability^34^. To enhance credibility and dependability, results were discussed with members of the midwifery core team. Code and theme names as well as citations were translated into English and translations were checked by a German and an English native speaker. Qualitative data were analyzed using Atlas.ti (Version 8.0).

## RESULTS

### Characteristics of participants

A total of 43 midwives working in the University hospital of Zurich participated in the survey, of whom 19 completed all three surveys. The median age of participants was 33.5 years (range: 25.0–64.0) (Table 1). Two-thirds of the midwives (n=28; 66.7%) were born in Switzerland, and half of the midwives (n=7; 50.0%) who were born abroad had lived 15 years and more in the country. The majority of the participants had a Bachelor’s degree (n=24; 57.1%) but 13 participants (31.0%) completed vocational training with midwifery diploma before the change of higher education according the Bologna process. The midwives had worked for a median of 9.2 years (range: 0.2–43.0) in the profession.

**Table 1:** Characteristics of participants, university hospital, Switzerland 2018 (N=43)

*Characteristics*	*Participants (N=42)^a^ n (%)*
**Age** (years), median (range)	33.5 (25–64)
**Country of birth**	
Switzerland	28 (66.7)
Germany	6 (14.3)
Serbia	2 (4.8)
Italy	1 (2.4)
Other	5 (11.9)
**Living in Switzerland (years)**	
<5	3 (21.4)
5–14	4 (28.6)
15–24	2 (14.3)
25–34	3 (21.4)
≥35	2 (14.3)
**Education level**	
Vocational training	13 (31.0)
Bachelor’s degree	24 (57.1)
Master’s degree	5 (11.9)
**Work years in health sector,** median (range)	14.5 (4.0–43.0)
**Work years as a midwife,** median (range)	9.2 (0.2–43.0)
**Work years at USZ^b^,** median (range)	9.5 (0.2–37.5)
**Workload (% of full time equivalent)**	
<50	1 (2.3)
50–70	12 (27.9)
80–100	30 (69.8)

a Missing values for one participant;

b USZ: University Hospital of Zurich.

### Job situation

Midwives indicated caring for a median of three women during one shift. Six midwives (14.3%) stated they sometimes or often needed to work overtime; for the others (n=36, 85.7%), this was rarely or never the case. All the midwives who indicated working overtime rarely, sometimes or often (n=24; 100%) were able to record these extra hours. Almost half of them (n=11; 45.8%) could compensate time during the same or the subsequent month (n=15; 62.5%) and two participants (8.3%) mentioned that overtime was paid (multiple answers allowed). Nearly all midwives (n=37; 97.4%) worked in shifts and most of them (n=29; 78.4%) worked all shifts (early, late and night shift). In a median, participants worked five-night shifts per month (range: 0–6).

### Attitude toward telephone debriefing

More than half of the midwives had an open attitude towards the telephone debriefing sessions before their implementation (54.3% absolutely or mostly agreed at t_0_, Table 2). There were no significant changes between the times of assessment (p=0.349). The proportion of midwives recognizing the telephone debriefing sessions as an additional stress increased slightly without significant difference between t_0_, t_1_ and t_2_ (p=0.469). However, participants acknowledging the project being important for the women decreased significantly between t_0_ and t_2_ (p=0.035).

**Table 2:** Attitude toward telephone debriefing before and after the implementation of the project, university hospital, Switzerland 2018 (N=43)

*Attitudes*	*Before implementation of debriefing sessions (t_0_) (n=35) n (%)*	*Two months after implementation of debriefing sessions (t_1_) (n=30) n (%)*	*Seven months after implementation of debriefing sessions (t_2_) (n=30) n (%)*
**I have an open attitude towards the telephone debriefing sessions**			
Absolutely	9 (25.7)	11 (36.7)	10 (33.3)
Mostly	10 (28.6)	10 (33.3)	8 (26.7)
Partly	14 (40.0)	8 (26.7)	11 (36.7)
Not at all	2 (5.7)	1 (3.3)	1 (3.3)
**Contacting women at home is an additional stress for me**			
Absolutely	9 (25.7)	9 (30.0)	8 (26.7)
Mostly	8 (22.9)	5 (16.7)	11 (36.7)
Partly	10 (28.6)	11 (36.7)	7 (23.3)
Not at all	8 (22.9)	5 (16.7)	4 (13.3)
**Debriefing sessions with the midwife who attended birth are important for women**			
Absolutely	11 (31.4)	5 (16.7)	2 (6.7)^a^*
Mostly	15 (42.9)	15 (50.0)	15 (50.0)
Partly	8 (22.9)	10 (33.3)	13 (43.3)
Not at all	1 (2.9)	0	0

a Significant difference between t_0_ and t_2_.

* p<0.05.

### Development of job satisfaction of midwives

The midwife-specific instrument to assess job satisfaction from Turnbull et al.^24^ showed a decrease and subsequently increases in the mean of the ‘Professional satisfaction subscale’ between t_0_, t_1_ and t_2_ from 0.77 to 0.66 to 0.81, respectively (Table 3). Regarding the ‘Professional support subscales’, mean scores also first decreased and subsequently increased without reaching the values of t_0_ (0.63 vs 0.37 vs 0.46). The mean scores of the ‘Client interaction subscale’ in contrast, increased slightly between t_0_ and t_1_ and more clearly between t_1_ and t_2_ (-0.06 vs -0.03 vs 0.17). Similarly, for the ‘Professional support’ subscales, the means of the ‘Professional development subscale’ first decreased and subsequently increased without reaching the value of t_0_ (0.72 vs 0.44 vs 0.52).

**Table 3:** Job satisfaction^a^ before and after the implementation of the project, university hospital, Switzerland 2018 (N=43)

*Job satisfaction (Coding: -2 to +2)*	*Before implementation of debriefing sessions (t_0_) (n=35) mean (SD)*	*Two months after implementation of debriefing sessions (t_1_) (n=30) mean (SD)*	*Seven months after implementation of debriefing sessions (t_2_) (n=30) mean (SD)*
**Professional satisfaction subscale**			
Generally speaking, I am satisfied with my current role as a midwife	1.34 (0.76)	1.10 (0.76)	1.30 (0.53)
I feel I am in a rut^b^	0.17 (1.27)	0.23 (1.14)	0.47 (1.01)
I feel frustrated with my current role^b^	0.91 (0.92)	0.73 (0.83)	0.97 (0.67)
I have enough opportunities to make decisions about care	0.20 (0.93)	0.13 (0.78)	0.40 (0.77)
I have limited opportunities for professional development^b^	0.46 (1.17)	0.33 (1.03)	0.13 (1.04)
I am confident that I have the skills for my current role	1.54 (0.78)	1.43 (0.57)	1.60 (0.56)
Mean professional satisfaction	0.77 (0.59)	0.66 (0.56)	0.81 (0.43)
**Professional support subscale**			
I have enough time to give women the care they need	0.14 (0.94)	-0.13 (1.14)	0.17 (0.91)
I get professional support from my midwife colleagues	1.46 (0.56)	1.23 (0.43)	1.33 (0.48)
I get enough support from other clinical colleagues (e.g. GPs and obstetricians)	0.63 (1.00)	0.47 (1.04)	0.73 (0.74)
There is not enough time to do my job properly^b^	0.83 (1.12)	0.43 (0.90)	0.33 (1.15)
My current role is very stressful^b^	0.11 (1.02)	–0.17 (0.79)	-0.27 (0.74)
Mean professional support	0.63 (0.55)	0.37 (0.50)	0.46 (0.52)
**Client interaction subscale**			
My current role allows me to provide women with choice about their care	0.29 (0.96)	0.20 (0.85)	0.43 (0.86)
My current role allows me to plan care with women	0.51 (0.85)	0.50 (0.86)	0.53 (0.78)
I need greater scope to provide women with information about their care^b^	-0.46 (0.89)	-0.33 (0.99)	0.10 (0.92)
I have limited opportunities to provide women with individualized care^b^	-0.17 (1.01)	-0.20 (0.89)	0.07 (0.91)
I have limited opportunities to provide continuity of care^b^	-0.46 (0.98)	-0.30 (0.88)	-0.27 (0.87)
Mean client interaction	-0.06 (0.76)	-0.03 (0.55)	0.17 (0.64)
**Professional development subscale**			
I have enough professional independence	0.26 (1.01)	–0.13 (0.90)	0.03 (0.89)
I have few opportunities to develop my skills as a midwife^b^	0.74 (1.04)	0.83 (0.91)	0.57 (1.07)
I have plenty of opportunities to further my professional education	0.89 (0.96)	0.83 (0.87)	0.43 (1.01)
I lack professional support from my managers^b^	1.49 (0.89)	1.20 (1.10)	1.57 (0.77)
Mean professional development	0.72 (0.70)	0.44 (0.66)	0.52 (0.55)

a Turnbull et al.^24^.

b Negative questions, which were recoded.

Higher values signify higher satisfaction.

**Figure 1:**
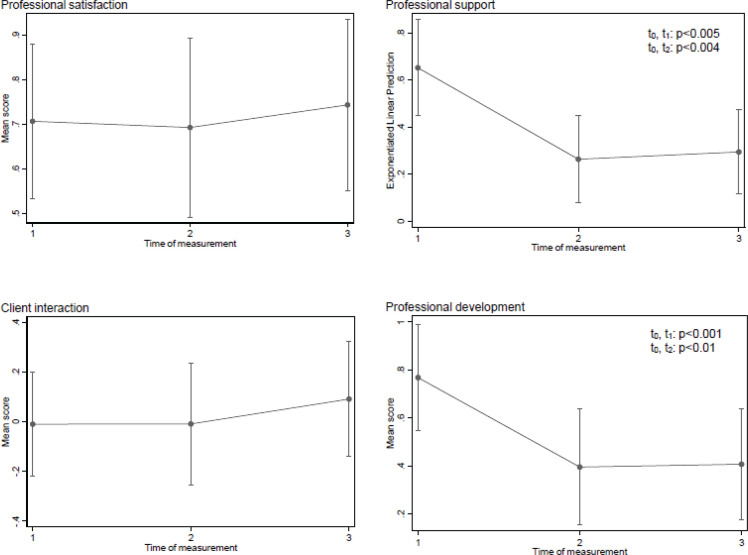
Adjusted trajectory of sub scores over measures

Repeated measure prediction for scores of subscales were adjusted for age, work years in the institution, number of women cared for per shift and workload. The adjusted predicted scores of the ‘Professional satisfaction subscale’ neither differed significantly between t_0_ and t_1_ (0.71; 95% CI: 0.53–0.88 vs 0.69; 95% CI: 0.49–0.89, p=0.906) nor between t_0_ and t_2_ (0.71; 95% CI: 0.53–0.88 vs 0.74; 95% CI: 0.55–0.94, p=0.745) (Figure 1). In contrast, the adjusted predicted scores of the ‘Professional support subscales’ declined significantly between t_0_ and t_1_ (0.65; 95% CI: 0.45–0.86 vs 0.26; 95% CI: 0.08–0.45, p=0.005) and t_0_ and t_2_ (0.65; 95% CI: 0.45–0.86 vs 0.29; 95% CI: 0.12–0.47, p=0.004). Regarding the ‘Client interaction subscale’, the adjusted predicted scores did not differ significantly either between t_0_ and t_1_ (-0.01; 95% CI: -0.22–0.20 vs -0.01; 95% CI: -0.25–0.23, p=0.995) or between t_0_ and t_2_ (-0.01; 95% CI: -0.22–0.20 vs 0.09; 95% CI: -0.14–0.32, p=0.460). A significant decrease was also observed for the adjusted predicted scores of the ‘Professional development subscale’ between t_0_ and t_1_ (0.77; 95% CI: 0.55–0.99, vs 0.40; 95% CI: 0.15–0.64, p<0.001) as well as t_0_ and t_2_ (0.77; 95% CI: 0.55–0.99 vs 0.41; 95% CI: 0.17–0.64, p<0.01).

### Other job and occupational related factors

The adjusted predicted scores of the ‘Autonomy subscale’ of LQWLQ-N did not significantly differ between the times of assessments before and after the implementation of the telephone debriefing sessions (t_0_ and t_1_, p=0.272; t_0_ and t_2_, p=0.125) (Table 4). Adjusted predictions for the ‘Meaning of work’ subscale of the COPSOQ was significantly lower at t_1_ compared to t_0_ (80.8; 95% CI: 75.7–85.9 vs 87.4; 95% CI: 82.9–91.9, p=0.014) but not between t_0_ and t_2_ (87.4; 95% CI: 82.9–91.9 vs 83.9; 95% CI: 79.0–88.8, p=0.178). Scores of the subscale ‘Bonding with the organization’ in contrast did not show significant differences either between t_0_ and t_1_ (p=0.272) or between t_0_ and t_2_ (p=0.691). Similar pictures were seen for the score of the ‘Work privacy conflict’ subscale (t_0_ and t_1_: p=0.580; t_0_ and t_2_: p=0.911) as well as the ‘Demarcation’ subscale (t_0_ and t_1_: p=0.328; t_0_ and t_2_: p=0.139). Adjusted predictions for the ‘Job satisfaction’ subscale were slightly but not significantly lower at t_1_ compared to t_0_ (63.4; 95% CI: 58.3–68.4 vs 65.7; 95% CI: 61.1–70.2, p=0.315) but showed a significant decrease between t_0_ and t_2_ (65.7; 95% CI: 61.1–70.2 vs 60.2; 95% CI: 55.4–65.1, p=0.017). While the adjusted ‘Intention to leave the organization’ subscale scores were similar at all times of assessment (t_0_ and t_1_: p=0.731; t_0_ and t_2_: p=0.291), the ‘Intention to leave the profession’ subscale differed between t_0_ and t_2_ (11.3; 95% CI: 5.9–16.7 vs 17.5; 95% CI: 11.5– 23.4, p=0.041) but not between t_0_ and t_1_ (11.3; 95% CI: 5.9–16.7 vs 9.8; 95% CI: 4.0–15.6, p=0.606)

**Table 4:** Results of adjusted work-related assessments before and after the implementation of the project, university hospital, Switzerland 2018 (N=43)

*Subscales*	*Before implementation of debriefing sessions (t_0_) (n=35) AP (95% CI)*	*Two months after implementation of debriefing sessions (t_1_) (n=30) AP (95% CI)*	*Seven months after implementation of debriefing sessions (t_2_) (n=30) AP (95% CI)*
**LQWLQ-N^a^**			
Subscale ‘Autonomy’	2.6 (2.6–2.8)	2.6 (2.4–2.7)	2.5 (2.4–2.7)
**COPSOQ** ^b^			
Meaning of work	87.4 (82.9–91.9)	80.8 (75.7–85.9)	83.9 (79.0–88.8)^c^*
Bond with organization	65.3 (59.9–70.6)	61.7 (55.7–67.8)	64.0 (58.2–69.8)
Work privacy conflict	34.7 (28.3–41.0)	36.3 (29.3–43.2)	34.4 (27.7–41.0)
Demarcation	26.5 (20.1–32.9)	30.1 (22.9–37.4)	20.8 (13.9–27.7)
Job satisfaction	65.7 (61.1–70.2)	63.3 (58.3–68.4)	60.2 (55.4–65.1)^d^*
Intention to leave the organization	17.1 (11.0–23.2)	18.4 (11.5–25.3)	21.1 (14.4–27.8)
Intention to leave the profession	11.3 (5.9–16.7)	9.8 (4.0–15.6)	17.5 (11.5–23.4)^d^*

AP: adjusted predictions.

a LQWLQ-N: Self-translated Leiden Quality of Work Life Questionnaire for Nurses.

b COPSOQ: Copenhagen Psychosocial Questionnaire (AP: 0 = ‘to a very small extent’ to 100 = ‘to a very large extent’).

c Significant adjusted difference between t_0_ and t_1_.

d Significant adjusted difference between t_0_ and t_2_.

* p<0.05.

### Results from focus group discussions

A total of eleven midwives participated in one or two of the three focus group discussions, seven in the first, five in the second and seven in the third. On average, midwives were aged 36 years (range: 26–52) and had 10 years of professional experience as a midwife (range: 1–30). The focus group discussions revealed four themes: ‘General job satisfaction’, ‘Challenges with the implementation’, ‘Continuity of care’ and ‘Meaning for the mothers’.

The theme ‘General job satisfaction’ comprised general aspects. Most midwives mentioned being very satisfied with their job and some of them linked their working years in the institution with their satisfaction. Having a secure job and working in a good team for example were considered as very important factors of job satisfaction:

*‘I mean … for me, the years indicate it (comment authors: the job satisfaction). If I was not be satisfied (laughs) … I would not be here anymore, definitively.’* (Before the implementation)

*‘And yes, I think as well… I experience that we are a really good team and help each other…’* (Before the implementation)

Factors negatively affecting job satisfaction were working shifts but also the request to complete an increasing number of tasks, which was felt to be insufficiently rewarded by the superiors. A trend toward negative quotations was higher at two and seven months after the implementation of the debriefing session, when interviews were conducted during periods with high workloads.

*‘Working shifts makes it nearly impossible to work 100 percent. Thus, working several years full time and keep the social environment intact is not possible.’* (Two months after the implementation)

*‘I think that employees must fulfil more and more tasks. We currently have the telephone debriefings and another project and I have the feeling that always more is demanded…’* (Two months after the implementation)

Regarding the ‘Challenges of the implementation’, midwives highlighted the additional workload of conducting the telephone debriefing sessions, which should be completed during their working time. They experience it as stressful, but even so, some of them point out the good feeling it gives them afterwards.

*‘And I have so many additional tasks (…). Thus … this extra task is stressful for me. I have always more tasks that I should manage.’* (Before the implementation)

*‘Once I've done it, I'm happy about it, but it is the same as for her (comment authors: name of the colleague), that it is always breathing down my neck.’* (Two months after the implementation)

The organization of the telephone debriefing sessions was especially challenging. Women were difficult to reach, and several calls or emails were needed to make contact.

*‘Yes, for me, this (comment author: the debriefing sessions) is really difficult to plan, this is a major challenge for me.’* (Seven months after the implementation)

‘Continuity of care’ was a controversial issue. Many participating midwives worked in a university hospital because they did not wish to be on call and wanted demarcation from work. However, seven months after the implementation of the telephone debriefing sessions, some midwives came to appreciate the follow-up contact with the women, which rounded off their care.

*‘Well, we are in a university hospital and probably have more women giving birth than in other places. And as already mentioned, some of us choose deliberately not to care for women during pregnancy, birth and the postpartum period but just to attend labor and birth.’* (Before the implementation)

*‘I think, this is also a lovely moment (…) we are taking care of one woman after the other (…) And then, I'm coming back again after two, three months (…) This is nice for me, that work is not already finished but we return to the birth situation.’* (Seven months after the implementation)

The participating midwives discussed intensely, but controversially, the ‘Meaning for the mothers’. On one hand, they recognized the benefit for mothers if they could clarify open questions about labor and birth. However, some midwives also questioned the necessity to call all women and thought that they could evaluate themselves if a woman needed a debriefing session or not.

*‘And she wanted to have confirmation that everything was completely normal and there was nothing bad, because she felt so harassed after having given birth. She experienced birth as an enormous event which ran over her body. She completely lost control and wanted to hear how I had experienced it.’* (Two months after the implementation)

*‘I mean, if the postpartum period is aggravated by uterine atony for example, then I understand it. But if everything is normal, I think we can assess it (comment author: if a debriefing session is necessary), in my experience.’* (Seven months after the implementation)

### Synthesis of results

Scores of job satisfaction, professional support and professional development decreased during the study period and slightly increased again towards the end. The intention to leave the profession simultaneously increased. The focus group discussions provided some explanations for these findings, such as additional stress because of the project but also because of higher workload due to increased birth rates at the second and third time of assessment. This showed the multifactorial character of job satisfaction and the impossibility to assess cause-effect conclusively. The third focus group discussion revealed differences between participants; some of them liked the telephone calls very much and did not want to stop them whereas others still did not see a sense in them. Seven months after the implementation of the telephone debriefing sessions, it remained unclear whether some midwives would need more time to integrate the telephone calls into their work routine. It might also have been important for them to know the benefits for the women.

## DISCUSSION

This is the first study investigating job satisfaction of midwives in the context of a self-initiated and self-responsible project to enhance continuity of care for midwives working in a medical-led maternity unit. Quantitative and qualitative data revealed that additional tasks due to the implementation of the project increased work-related stress in the short-term. Additional stress seems to have a stronger effect on job satisfaction than increased self-responsibility and the opportunity to deepen the relationship with women.

Despite being initiated by a midwifery core group and fostering self-responsible work as well as continuity of care, our study did not show an increase in job satisfaction but a decrease in the first phase. This was in contrast to studies investigating job satisfaction of midwives working in midwife-led models of care, where self-responsibility and continuity of care might be causes for the increased satisfaction^14,16^. However, the changes in the work situation in our project were less substantial than in midwife-led care as medical-led care was still dominant. Nevertheless, qualitative data showed that some midwives appreciated increasingly the additional contact with women during the postpartum period. They indicated that this had an impact on their relationship with parturients and intrapartum care. This was in line with findings of an Australian study which reported that a positive factor of continuity of care was the opportunity to build relationships^15^.

It can be assumed that the additional tasks in the current study lead to an increased workload and decreased satisfaction. This confirmed the results of other studies that workload is a potential work-related stressor for midwives^11,13,32^. The results of the current study were also consistent with those of one which showed dissatisfaction in situations where additional tasks led to a very high workload^35^.

The longitudinal character of our study might have added the development of job satisfaction in a very early phase of the implementation of a project as a new aspect. Two months after starting the telephone debriefing sessions, the organizational aspects had priority and many midwives were not able to acknowledge the benefits of the project. Additionally, the qualitative findings indicated that the project has probably not been accepted by all midwives. This might be the cause of the initial decrease and subsequent increase of job satisfaction during the study period. It is also a known phenomenon that healthcare providers attitude can be a hindering contextual factor in implementation studies^36^. The critical attitude of some midwives towards the project could have increased the perception of additional stress. Midwives might have needed time to accept the project and recognize its benefits. A Danish study showed that providing high-quality care led to increased job satisfaction^10^. Interviews with the users of the telephone debriefing sessions in our study, which have been published elsewhere, emphasized the satisfaction of women and the benefits of processing birth^37^. However, these results were not known until the end of the project and only had a minimal impact on the third group discussion. It is possible that it was too early to assess the long-term development of job satisfaction seven months after the implementation of the debriefing sessions. Nevertheless, improving the working environment of midwives is very important to increase job satisfaction and prevent early career leavers and skill shortage^2,3,5^. Future study should plan longer and more frequent follow-up sessions and provide opportunities to discuss the additional knowledge about the benefits for women.

### Strengths and limitations

Strengths of our study were the use of questions from validated instruments combined with qualitative data from focus group discussions. Quotations of midwives provided a deeper insight leading to explanations for some quantitative findings. Nevertheless, there was limited space to present the qualitative results in detail. Furthermore, the small sample size, which was due to the limited number of midwives working in the same hospital, is a limitation. Additionally, the single center study might not have provided results which can be generalized to other settings. Midwives working in larger maternity units might not have the same interest in continuity of care as those working in smaller ones. Due to the relatively large team with a rather hierarchical structure, the project was developed by a core team. This may have prevented all midwives from identifying with the project. During the study phase, which lasted nearly one year, staff turnover was observed causing incomplete follow-up data, as well as new participants. In addition, there were some midwives who did not participate in the study for unknown reasons (14.0%). It cannot be ruled out that some of them expressed their rejection of the project by not participating in the survey. The Generalized Estimating Equation models, however, allowed the inclusion of information of all participants with or without complete data. However, natural job fluctuation and dropout resulted in an incomplete data set. While informal assessment of missing cases at various time points did not suggest systematic attrition, unbalanced data is a potential limitation of the study. Additionally, the chances of changing external factors such as periods with higher and lower workloads or new regulations were also increased due to the duration of the study.

## CONCLUSIONS

Our study showed a decreased job satisfaction in the early phase of a new project. The effects of increased work-related stress because of additional tasks were stronger than effects of acquiring more responsibility and have a deeper relationship with clients because of enhanced continuity of care. This knowledge is important for heads of institutions and policy makers because strategies to support midwives during implementation phases and additional resources might be necessary to prevent decreased job satisfaction. It remained unclear how satisfaction of midwives would develop during a longer time period. Future studies should investigate job satisfaction in the context of self-initiated and self-responsible projects in larger samples and different settings and consider a longer follow-up.

## Data Availability

The data supporting this research are available from the authors on reasonable request.
